# Association of SARS susceptibility with single nucleic acid polymorphisms of OAS1 and MxA genes: a case-control study

**DOI:** 10.1186/1471-2334-6-106

**Published:** 2006-07-06

**Authors:** Jing He, Dan Feng, Sake J de Vlas, Hongwei Wang, Arnaud Fontanet, Panhe Zhang, Sabine Plancoulaine, Fang Tang, Lin Zhan, Hong Yang, Tianbao Wang, Jan H Richardus, J Dik F Habbema, Wuchun Cao

**Affiliations:** 1 Beijing Institute of Microbiology and Epidemiology, State Key Laboratory of Pathogen and Biosecurity, Beijing, China; 2 Department of Public Health, Erasmus MC, University Medical Center, Rotterdam, The Netherlands; 3 Emerging Diseases Epidemiology Unit, Institut Pasteur, Paris, France; 4 Université René Descartes, INSERM U.550, Faculté de Médecine Necker, Paris, France; 5 Beijing General Hospital of Armed Police, Beijing, China

## Abstract

**Background:**

Host genetic factors may play a role in susceptibility and resistance to SARS associated coronavirus (SARS-CoV) infection. The study was carried out to investigate the association between the genetic polymorphisms of 2',5'-oligoadenylate synthetase 1 (OAS1) gene as well as myxovirus resistance 1 (MxA) gene and susceptibility to SARS in Chinese Han population.

**Methods:**

A hospital-based case-control study was conducted. A collective of 66 SARS cases and 64 close contact uninfected controls were enrolled in this study. End point real time polymerase chain reaction (PCR) and PCR-based Restriction Fragment Length Polymorphism (RFLP) analysis were used to detect the single nucleic polymorphisms (SNPs) in OAS1 and MxA genes. Information on other factors associated with SARS infection was collected using a pre-tested questionnaire. Univariate and multivariate logistic analyses were conducted.

**Results:**

One polymorphism in the 3'-untranslated region (3'-UTR) of the OAS1 gene was associated with SARS infection. Compared to AA genotype, AG and GG genotypes were found associated with a protective effect on SARS infection with ORs (95% *CI*) of 0.42 (0.20~0.89) and 0.30 (0.09~0.97), respectively. Also, a GT genotype at position 88 in the MxA gene promoter was associated with increased susceptibility to SARS infection compared to a GG genotype (*OR *= 3.06, 95% *CI*: 1.25~7.50). The associations of AG genotype in OAS1 and GT genotype in MxA remained significant in multivariate analyses after adjusting for SARS protective measures (*OR *= 0.38, 95% *CI*: 0.14~0.98 and *OR *= 3.22, 95% *CI*: 1.13~9.18, respectively).

**Conclusion:**

SNPs in the OAS1 3'-UTR and MxA promoter region appear associated with host susceptibility to SARS in Chinese Han population.

## Background

Severe acute respiratory syndrome (SARS) is an emerging infectious disease caused by a novel positive-stranded RNA virus, termed SARS-CoV [1]. The global outbreak of SARS has killed 774 and infected more than 8098 people in 29 countries and regions, as reported by WHO [2]. In Beijing, more than 2523 cases and 181 deaths occurred including health care workers and family members who had closely contacted with SARS patients [3]. It was noticed that despite similar levels of exposure to the SARS-CoV some people were infected while others were still free from infection, suggesting a role of host genetic factors in susceptibility to SARS-CoV infection [4]. Human-Leukocyte-Antigen Class I and Class II Genotypes were previously reported to be associated with the development of SARS [5,6]. The polymorphism of angiotensin converting enzyme 1 gene was supposed to be involved in the progression of the disease [7].

Resent study showed that type I interferons (IFN-c/d) could inhibit SARS-CoV replication both in *vivo *and in *vitro *[8-10]. Type I interferons induce numerous proteins with antiviral activities, including 2'-5' oligoadenylate synthetase 1 (OAS1) and myxovirus resistance 1 (MxA). OAS1 protein can bind double-stranded RNA and polymerize ATP into PPP (A2'P5'A) N oligomers, which activate the latent RNAse L that, when activated, cleave single-stranded RNAs [11]. MxA protein belongs to the dynamin family and shows activity against several viruses [12-14]. However, the precise mechanism of antiviral action has not been elucidated. Host genetic factors that influence antiviral effects of interferons were well demonstrated in viral hepatitis, especially of hepatitis C virus infection [15]. It was reported that the polymorphisms at position 347 in the 3'-untranslated region (3'-UTR) of the OAS1 gene and at position 88 in the MxA gene promoter region seemed associated with hepatitis C virus infection [16]. The polymorphisms of these two genes that might affect the susceptibility to SARS were reported in Vietnamese population [17]. The G allele frequencies of OAS1 at the position 347 in the 3'-UTR were estimated to be 41.7% among European-Americans, 10.9% among African-Americans, 18.2% among Japanese and 36.4% among Chinese. The T allele frequencies of MxA at position 88 in the promoter region were estimated to be 19.6% among Caucasian and 33% among Vietnamese population. The objective of the study was to investigate if single nucleic acid polymorphisms (SNPs) of OAS1 and MxA genes were associated with the development of SARS in Chinese Han Population.

## Methods

### Cases and controls

A total of 66 confirmed SARS cases were included in the study. All cases were Chinese Hans from a designated hospital for SARS in Beijing. They were diagnosed according to the criteria published by Chinese Ministry of Public Health, and subsequently confirmed by serological test.

All the controls were also Chinese Han people. They were healthy doctors and nurses working in the same hospital, with a history of close contact with SARS patients. If a person had shared a meal, utensil, residence, ward, vehicle etc. with a SARS patient or visited a patient, a contact history of the person was realized. In addition, contagium was also considered as close contact. All the controls were negative for IgG antibody against SARS-CoV. Any controls who are consanguineous with any cases were excluded.

### Information collected using a questionnaire

A questionnaire was developed to collect information from both cases and controls on demographic characteristics (age, gender, region of origin, etc.), habits and medical history (smoking and drinking habit, diet, exercise, medical history, history of operations, etc.), and protective measures (wearing masks, gowns and goggles when in contact with SARS patients). All the cases and controls filled in the questionnaire before providing 5 ml anti-coagulated blood with EDTA. All participants signed an informed consent. The study protocol was approved by the ethical committee at National Center for AIDS Prevention and Control.

### DNA extraction

Leucocytes were isolated within 12 h of blood collection using Percoll reagent. Then genomic DNA was extracted using cell DNA extraction kit (BioDEV Inc. Beijing, China) according to the manufacturer's instructions.

### Genotyping of allelic variants of the *OAS1 *and *MxA *genes

The OAS1 gene's A/G SNP at the 3'UTR 347 locus of the exon 8 (*GenBank Acc No. *M11810) was investigated in the study. End-point real time PCR method was used in the study. The Assays-on-Demand SNP Genotyping Products kit was purchased from Applied Biosystems. Briefly, the TaqMan Universal PCR Master Mix reagent containing a pair of unlabeled primes and two allele-specific probes were designed according to the SNP at 347 A/G 3' UTR of the *OAS1 *gene (NCB1 SNP Acc No.r s2660). One probe labeled FAM contains the A allele, and the other probe labeled VIC contains G allele. PCR amplifications and fluorescent detections were performed in an ABI Prism 7900HT sequence detection system (ABI Ltd.). PCR mixture in 25 μl volumes contained 20 ng genomic DNA, 2 × TaqMan Universal PCR Master Mix No AmpErase UNG (12.5 μl), 20 × Assays-on-Demand SNP Genotyping Assay Mix (1.25 μl). The cycling protocol was as follows: 10 min at 95°C, followed by 40 cycles of 92°C for 15 s, 60°C for 1 min. The fluorescent signals were detected after the amplification. The sample with high red fluorescent signals was AA genotype, the sample with high green fluorescent signals was GG genotype, the sample with both high fluorescent signals was AG genotype. Each sample was detected two or three times.

The allelic G/T polymorphism in the promoter region of MxA at position 88 from the transcription start site was genotyped by PCR-restriction fragment length polymorphism (RFLP) method as previously described [16]. Amplification was carried out in a volume of 20 μl, containing 10 – 100 ng DNA, 2.5 mM MgCl2, 500 nM of each primer, 500 mM dNTP's, 1 × PCR buffer, 2 U Taq DNA polymerase (Shanghai Sangon Biological engineering technology and service Ltd., China). The cycling conditions in an Applied Biosystems 2400 machine were: denaturation at 94°C for 5 min, subsequently 35 cycles of denaturation at 94°C for 30 s; annealing at 58°C for 30 s; and extension at 72°C for 1 min. This was followed by a final extension at 72°C for 7 min. A total of 8 μl of the PCR product was digested for 4 h in a volume of 20 μl with 5 U HhaI according to the manufacturer's instruction. A volume of 10 μl digested PCR product was separated on 2% agarose gels and visualized under ultraviolet light. In the presence of the G allele, the 351-bp PCR product was cut into 4 fragments (261, 51, 23 and 16 bp, respectively), and in the presence of the T allele into 3 fragments (312, 23 and 16 bp, respectively).

### Statistical analysis

Data were analyzed using the SPSS software (version 10.0, SPSS Inc, Chicago, IL, USA). Unconditional logistic regression was performed to estimate odds ratios (ORs) and their 95% confidence intervals (*CI*). Univariate analyses were conducted to determine the effect of each variable separately. Multivariate analyses were then performed using genetic polymorphisms as independent variables and all the significant variables in univariate analyses as covariables. For all the analyses, statistical tests were based on two-tailed probability. A *P *value <0.05 was considered statistically significant.

## Results

### Demographic characteristics of cases and controls

A total of 66 cases and 64 controls were included in the study. The mean age was 28.3 years for cases and 29.4 years for controls (*P *> 0.05). The proportion of males was 48.5% in cases and 68.8% in controls (*P *> 0.05).

### Univariate analysis results of factors related to SARS

Several protection measures including wearing masks, gowns and goggles were found to be significantly associated with decreased risk of SARS-CoV infection (*P *< 0.05). No significant difference between cases and controls was observed for the other factors, including smoking, alcohol-drinking, nutrition status and physical activities (Table 1).

**Table 1:** Distribution of SARS-related factors in cases and controls

Variables	Cases (%)	Controls (%)	*P *value
Wearing a mask			0.003
Always	38 (57.6)	54 (84.4)	
Occasionally	12(18.2)	6(9.4)	
Never	16(24.2)	4(6.2)	
Wearing a gown			<0.001
Always	21(31.8)	50(78.1)	
Occasionally	2(3.0)	2(3.1)	
Never	43(65.2)	12(18.8)	
Wearing goggles			<0.001
Always	18(27.3)	52(81.3)	
Occasionally	7(10.6)	5(7.8)	
Never	41(62.1)	7(10.9)	
Smoking			0.434
Yes	7(11.1)	10(15.9)	
No	56(88.9)	53(84.1)	
Alcohol-drinking			0.914
Never	35(53.8)	33(53.2)	
Occasionally	26(40.0)	24(38.7)	
Often	4(6.2)	5(8.1)	
Meat intake			0.616
Every day	34(51.5)	34(53.1)	
Often	29(43.9)	29(45.3)	
Rarely	3(4.6)	1(1.6)	
Physical exercise			0.213
Rarely	18(28.1)	20(31.7)	
1~2 times/week	27(42.2)	34(54.0)	
>3 times/week	10(15.6)	5(7.9)	
Every day	9(14.1)	4(6.4)	

### Univariate analysis results of OAS1 and MxA gene SNPs in relation to SARS

Allele frequencies of OAS1 and MxA genes in cases and controls were compared (Table 2). The AG and GG genotypes in the 3'UTR of the OAS1 gene were significantly less frequent in case group (33.3% and 7.6%, respectively) than in control group (48.4% and 15.6%, respectively). Compared to AA genotype, AG and GG genotypes were found associated with a protective effect on SARS with ORs (95% *CI*) of 0.42 (0.20~0.89) and 0.30 (0.09~0.97), respectively. Also, a GT genotype at position 88 in the MxA gene promoter was associated with increased susceptibility to SARS infection compared to a GG genotype (*OR *= 3.06, 95% *CI*: 1.25~7.50).

**Table 2:** Univariate analysis results of *OAS1 *AND *MxA *gene SNPs in cases and controls

	Cases (%)	Controls (%)	OR (95% *CI*)	*P *value
*OAS1 3'-*UTR				
genotype				0.026
AA	39 (59.1)	23 (36.0)	1.00	
AG	22 (33.3)	31 (48.4)	0.42 (0.20~0.89)	0.022
GG	5 (7.6)	10 (15.6)	0.30 (0.09~0.97)	0.038
allele				
A	75.8	60.2		
G	24.2	39.8		
*MxA *-88				
genotype				0.006
GG	13(19.7)	24(37.5)	1.00	
GT	48(72.7)	29(45.3)	3.06(1.25~7.53)	0.006
TT	5(7.6)	11(17.2)	0.84(0.20~3.44)	0.784
allele				
G	56.1	61.1		
T	43.9	38.9		

### Multivariate analysis results of OAS1 and MxA gene SNPs in relation to SARS

A multivariate analysis was conducted with disease status as the dependent variable, each genotype as an independent variable, and wearing masks, gowns and goggle as covariables. It was indicated that the individuals with OAS1 AG genotype were at decreased risk, and those with MxA genotype GT were at increased risk of contracting SARS infection, as compared to AA and GG for OAS1 gene, and to GG and TT for MxA gene, respectively (Table 3 and 4).

**Table 3:** Association between *OAS1 *genotypes and SARS infection after adjusting other related factors in multivariate analysis

Variable	OR (95% *CI*)	*P *value
Wearing a mask		0.230
Always	1.00	
Occasionally	1.70(0.42~6.86)	0.102
Never	3.11 (0.80~12.11)	0.456
Wearing a gown		0.490
Always	1.00	
Occasionally	1.23 (0.13~11.62)	0.234
Never	2.19 (0.60~8.00)	0.857
Wearing goggles		0.006
Always	1.00	
Occasionally	2.92(0.64~13.23)	0.002
Never	8.53 (2.16~33.73)	0.166
genotype		0.125
AA	1.00	
AG	0.38(0.14~0.98)	0.047
GG	0.46(0.11~1.96)	0.291

**Table 4:** Association between *MxA *genotypes and SARS infection after adjusting other related factors in multivariate analysis

Variable	OR (95% *CI*)	*P *value
Wearing a mask		0.100
Always	1.00	
Occasionally	1.64(0.41~6.60)	0.486
Never	4.41(1.12~17.34)	0.034
Wearing a gown		0.753
Always	1.00	
Occasionally	1.03 (0.10~10.75)	0.460
Never	1.63(0.45~6.01)	0.983
Wearing goggles		0.004
Always	1.00	
Occasionally	3.48(0.77~15.80)	0.106
Never	9.51(2.34~38.73)	0.002
genotype		0.061
GG	1.00	
GT	3.22 (1.13~9.18)	0.029
TT	1.17 (0.25~5.60)	0.843

## Discussion

Many factors may influence the outcome and clinical manifestations of SARS infection, such as exposure levels, differences in SARS-CoV strain virulence, host health status, smoking, other virus co-infection, and host genetic patterns. In order to study the association between gene polymorphisms and SARS-CoV infection, we took several measures to control for possible confounders. Firstly, cases and controls were selected from the same hospital to minimize the impact of virulence differences in SARS-CoV strains. Secondly, each control had to have close contact history with SARS patients to reduce differences in exposure levels between cases and controls. Thirdly, multivariate analyses were conducted to adjust the factors which were statistically significant in univariate analyses. In fact, mask-, gown- and goggles-wearing were found to be protective against SARS-CoV infection in this study as previously reported [18-20].

We found that both the AG and GG genotypes of OAS1 gene at the 3'-UTR region were more common among controls compared to cases. The G allele frequency among controls (39.8%) was comparable to that found in the 45 Han Chinese in Beijing. Our findings indicate that the G allele in the OAS1 gene conferred protection against SARS-CoV infection. Knapp reported that this A/G allele was associated with the outcome of HCV infection, OAS1 playing a greater role in mediating self-limiting versus persistent HCV infection, rather than when viral persistence is established [16]. A nonsense mutation described in the gene encoding the mouse 2'-5' OAS1 isoform was associated with West Nile virus susceptibility in laboratory mice [21,22]. There are two isoforms in human OAS1 gene (E16 and E18), which share identical N-terminal sequence but diverge at exon 7 [23]. Isoform E16 contains 7 exons and a hydrophobic C-terminus. Isoform E18 contains an additional exon and is very hydrophilic as compared to transcript variant E16. Production of the two mRNAs isoforms of OAS1 depends on processing that involves the recognition site located within the 3'-terminal exon [24]. The polymorphisms in the 3'UTR may be related with the expression of the two isoforms. The mechanisms of the protective effect of G allele need to be elucidated.

Regarding the G/T polymorphism at position 88 in promoter region of MxA gene, the GT genotype was found to be more frequent in patients than in controls. Other researchers found no difference in SARS-CoV replication in Vero cells that were stably expressing MxA [25,26]. The patients with GT genotype may express more MxA protein when induced by IFNs. Consequently, the MxA protein can promote the establishment of persistent SARS-CoV infection. MxA protein may have dual functions in reaction to virus infection. Torisu reported that T allele with a high MxA-producing capability was more frequently seen in subacute sclerosing panencephalitis (SSPE) patients [27]. A luciferase reporter assay revealed that the MxA promoter sequence of T haplotype had higher promoter activity than that of G haplotype [28]. Arcas *et al*. reported that GT and TT genotype expressed higher amount of MxA mRNA than GG genotype in IFN-treated peripheral blood mononuclear cells in *vitro *[29]. In our study, 88 SNP in MxA was related to disease susceptibility. The mechanism of the MxA gene with SARS susceptibility needs to be further investigated. Since the polymorphisms in the two genes were associated with the SARS-CoV infection, we tried to find out if combined action between mutations could affect the susceptibility to SARS-CoV infection. But the sample size of this study is not sufficient to do the interaction analysis. More SARS cases and controls are required to do so.

## Conclusion

In conclusion, we showed that the SNPs in OAS1 and MxA genes were associated with SARS-CoV infection in Chinese Han population. These findings may lead to a better understanding of IFN-induced antiviral response to SARS infection.

## Competing interests

The author(s) declare that they have no competing interests.

## Authors' contributions

HJ carried out the study design, OAS1 genotyping and drafted the manuscript. DF carried out study design and statistical analysis. SJDV participated in the study design, statistical analysis and helped to draft the manuscript. HW carried out the genotyping of MxA gene. PZ, FT, LZ and HY participated in the blood sample and information collection in the field, and carried out the DNA extraction. AF participated in the study design and helped to draft the manuscript. SP wrote the genotyping protocol. TW coordinated the blood sample and information collection. JHR and JDFH participate in the study design and refined the manuscript. WC conceived the study and coordinated all the activities.

## Pre-publication history

The pre-publication history for this paper can be accessed here:


